# Evaluating the impact of triple-drug mass drug administration using human and mosquito-based indicators of lymphatic filariasis in Samoa, 2019 – 2024

**DOI:** 10.1371/journal.pntd.0013895

**Published:** 2026-10-15

**Authors:** Helen J. Mayfield, Angus McLure, Lisa Rigby, Brady McPherson, Richard Bradbury, Shannon M. Hedtke, Rhys Izuagbe, Joanne Kizu, Donna MacKenzie, Elina Panahi, Beatris Mario Martin, Benn Sartorius, Asrhyella Seeler, Selina Ward, Robert Thomsen, Satupaitea Viali, Patricia M. Graves, Colleen L. Lau

**Affiliations:** 1 Faculty of Health, Medicine and Behavioural Sciences, University of Queensland Centre for Clinical Research, The University of Queensland, Brisbane, Queensland, Australia; 2 National Centre for Epidemiology and Population Health, Australian National University, Canberra, Australian Capital Territory, Australia; 3 Australian Defence Force Malaria and Infectious Disease Institute, Enoggera, Queensland, Australia; 4 College of Medicine and Dentistry, James Cook University, Cairns, Queensland, Australia; 5 Institute of Innovation, Science and Sustainability, Federation University, Berwick, Victoria, Australia; 6 La Trobe Institute for Molecular Science, School of Agriculture, Biomedicine and Environment, La Trobe University, Bundoora, Victoria, Australia; 7 QIMR Berghofer Institute of Medical Research, Brisbane, Queensland, Australia; 8 Samoa Ministry of Health, Apia, Samoa; 9 Oceania University of Medicine Samoa, Apia, Samoa; Washington University in St Louis School of Medicine, UNITED STATES OF AMERICA

## Abstract

**Background:**

For lymphatic filariasis (LF) elimination, the World Health Organization recommends multiple rounds of mass drug administration (MDA) to interrupt transmission. While LF antigen (Ag) is routinely used to monitor progress, recent evidence suggests more time-sensitive indicators are needed during the immediate post-MDA period. In Samoa, triple-drug MDA was distributed in 2018 and 2023. This study aimed to evaluate the impact of Samoa’s second round of triple-drug MDA on human and mosquito-based indicators of transmission.

**Methodology:**

Surveys were conducted in eight primary sampling units (PSUs) in 2019 (6–9 months after the 2018 MDA), 2023 (4.5 years after the 2018 MDA) and 2024 (10 months after the 2023 MDA). Participants aged ≥5 years from randomly selected households were tested for Ag and microfilariae (Mf). For molecular xenomonitoring (MX), mosquitoes were caught using BG-Sentinel traps at households, sorted into pools by species, and tested for presence of filarial DNA using quantitative polymerase chain reaction (qPCR).

**Results and key findings:**

In 2024, Ag prevalence was 10.3% (95% CI: 7.3-14.5) versus 9.9% (95% CI: 3.5-21.0) in 2023 and 9.8% (95% CI: 5.6-15.5) in 2019. Mf prevalence was 3.1% (95% CI: 1.8-5.5) in 2024 vs 5.1% (95% CI:1.3-12.4) in 2023 and 3.1% (95% CI:1.3-5.9) in 2019. Odds ratios (OR) of a positive test in 2024 vs 2023 showed no decrease for Ag (OR 1.0; 95% CI: 0.7-1.6), but a potential reduction in the proportion of Ag-positive participants who were Mf-positive (OR 0.6; 95% CI: 0.2-1.4) and a significant reduction in the prevalence of qPCR-positive mosquitoes (OR 0.4; 95% CrI: 0.2-0.7). From 2019–2024, there were reductions in prevalence of qPCR-positive *Aedes* spp. (OR 0.5; 95% CrI: 0.3-1.0) and *Aedes polynesiensis* (OR 0.4; 95% CrI: 0.2-0.8).

**Conclusions:**

In Samoa, LF transmission continues despite two rounds of triple-drug MDA five years apart. Mosquito indicators can provide a more sensitive measure of MDA impact in the immediate post-intervention period and complement human indicators for long-term surveillance.

## Author summary

Lymphatic filariasis is a mosquito-borne disease that can cause severe long-term disability. To eliminate this neglected tropical disease as a public health problem, the World Health Organization (WHO) recommends multiple rounds of mass drug administration (MDA) 12 months apart. Despite antigen (Ag) being the standard indicator for evaluating MDA impact, more time-sensitive indicators, such as measuring microfilariae (Mf) in the blood or testing mosquitoes for filarial DNA (molecular xenomonitoring), may provide a more reliable assessment in the months following the intervention. Samoa distributed triple-drug MDA in 2018 and 2023. We evaluated the impact of the second round on human and mosquito-based infection indicators including Ag, Mf, and qPCR-positive mosquitoes. We surveyed eight villages in Samoa in 2019 (6–9 months after the 2018 MDA), 2023 (4.5 years after the 2018 MDA) and 2024 (10 months after the 2023 MDA). The two rounds of MDA were insufficient to break the LF transmission cycle. Results from 2024 showed no decrease in Ag prevalence from either 2019 or 2023, although there was potentially a decrease in Mf prevalence. Molecular xenomonitoring (MX) provided an earlier signal of MDA impact than Ag, with a decrease in the prevalence of qPCR-positive mosquitoes observed within 10 months of the 2023 MDA.

## Introduction

Lymphatic filariasis (LF) is a mosquito-borne neglected tropical disease (NTD) that can cause debilitating lymphedema and scrotal hydroceles [1]. The disease is caused by infection from one of three parasites: *Wuchereria bancrofti*, *Brugia malayi,* and *Brugia timori*. The mosquito species that act as the vector vary from country to country and include species from the genera *Culex, Aedes, Anopheles,* and *Mansonia* [2]. Adult parasites reside and reproduce in the human lymphatic system, from which microfilariae (Mf) make their way into the circulating bloodstream, where they can be ingested by blood-feeding mosquitoes. Mf mature into their larval stages inside the thoracic muscles of mosquitoes before being transmitted to humans through a bite [3].

Despite decades of elimination efforts as part of the Global Programme to Eliminate LF (GPELF) [4], LF remains endemic in many parts of the world. As of 2024, 485.4 million people remain vulnerable to infection [5]. To achieve elimination of LF as a public health problem, WHO recommends multiple rounds of mass drug administration (MDA), delivered annually to endemic populations [6,7]. Since 2018, in certain settings where the standard two-drug regimen (albendazole plus diethylcarbamazine or ivermectin) has proven ineffective for reaching elimination as a public health problem, a triple-drug regimen combining all three drugs has been recommended [7].

The human-mosquito transmission cycle of the parasite allows programs to monitor progress towards elimination targets using human and/or mosquito-based indicators. For human-based indicators, this is most commonly achieved by testing blood samples for the presence of the antigen (Ag) produced by adult worms. This can be done easily using a rapid diagnostic test such as the Alere/Bioline Filariasis Test Strip (Scarborough, ME, USA) [8] or the more recently approved STANDARD Q Filariasis Ag Test (QFAT) [9,10]. Ag testing is often combined with microscopy of dried blood slides to detect circulating Mf [11]. Recently, the use of LF antibodies as a surveillance indicator has also been explored [3,12].

The use of molecular xenomonitoring (MX) for mosquito surveillance is becoming more common [13]. MX involves using a molecular diagnostic such as quantitative polymerase chain reaction (qPCR) to test individual or pools of mosquitoes for the presence of filarial DNA. This approach has been shown to be effective in detecting qPCR-positive mosquitoes in diverse settings such as French Polynesia [14], India [15], and American Samoa [16], as well as providing a sensitive indicator for detecting community-level changes in LF transmission in Samoa [17]. In Samoa, a study comparing an MX survey with a human Mf and Ag survey carried out at the same scale (survey time and sites covered) found that MX provided signals of transmission at more households than Mf screening [11]*.* MX studies in India [18] and Sri Lanka [19] have also demonstrated the superior sensitivity of MX surveillance-based indicators compared to human-based indicator*s.*

WHO criteria for a country to stop MDA are based on critical cut-offs of the number of Ag-positive people detected through Transmission Assessment Surveys (TAS). Specifically, where *Aedes* mosquitoes are the main vector, critical cut-offs are calculated based on the upper boundary of the 95% confidence interval for Ag prevalence in children aged 6–7 years being below 1% [6]. These thresholds are used to guide national programs to determine when transmission has been reduced to levels low enough that it is no longer considered sustainable. Of the 16 Pacific Islands and Territories where LF was endemic at the beginning of GPELF, eight have yet to be validated as having eliminated LF as a public health problem [20]. Following eight rounds of two-drug MDA between 1998 and 2017 [21], Samoa completed two rounds of triple-drug MDA in 2018 and 2023. Following the first round in 2018, which achieved a self-reported coverage of 80% [22], no significant change to Ag prevalence was observed after 6–8 months [23]. However, MX results revealed a decrease in the prevalence of mosquitoes at 7–9 months post-MDA [17]. The second triple-drug MDA in Samoa was delayed until September 2023 due to competing public health priorities (primarily a large measles outbreak [24] and COVID-19). At this time, Ag prevalence within these communities was not significantly different to those reported in 2019 [25].

MX is recommended as one of the four surveillance tools for post-validation surveillance of LF by WHO in the recent guidelines for monitoring and epidemiological assessment of MDA [6]. Before this advice can be practically applied in a standardised way, there is an urgent need to fill the gaps in our knowledge about how to use this tool efficiently and understand how survey results can be interpreted in relation to human-based indicators. Additionally, the lack of defined guidelines and stop-MDA targets for MX indicators limits the use of MX for guiding programmatic decisions. Here, we report the results from two MX community surveys in Samoa conducted in 2023 and 2024 (before and after the 2023 MDA). By comparing these results to Ag and Mf prevalence in samples collected during the same time period, with similar survey time and effort, and in the same communities, and comparing this to the previously reported Ag, Mf, and MX results from 2019 [17,23], this study aimed to evaluate the impact of triple-drug MDA on both human and mosquito-based indicators in Samoa. Specifically, the objectives were:

i To detect any evidence of ongoing LF transmission in Samoa after two rounds of triple-drug MDA distributed five years apart.
ii To evaluate the impact of the second round of triple-drug MDA (in 2023) on human (Ag, Mf) and mosquito (filarial DNA) indicators in eight primary sampling units 10 months after triple-drug MDA.
iii To compare the human and mosquito-based indicators for evaluating the impact of triple-drug regimen in Samoa.

## Methods

### Ethics

Ethics approvals were granted by the Samoa Ministry of Health and The University of Queensland Human Research Ethics Committee (protocols 2021/HE000895 and 2024/HE001263). The study was conducted in close collaboration with the Samoa Ministry of Health, the WHO country office in Samoa, and the Samoa Red Cross. Permission was sought from village leaders before entering a village. Verbal and written informed consent were obtained from all participants and from the parents or guardians of participants under the age of 18 years.

### Study region

Samoa, located in the South Pacific, is comprised of two main Islands, Upolu and Savai’i, with small populations on Manono and Apolima Islands. Samoa has a population of around 200,000 residents, with 78% residing on Upolu [26]. The country is predominately rural, with large forested areas. The majority of the population reside in small coastal villages or in the urban area around the capital Apia. Traditional housing (covered fales with no walls) is common in the rural and peri-urban areas. The primary LF vector species is *Aedes (Ae.) polynesiensis,* a highly efficient diurnal or day-biting mosquito that feeds and rests outdoors (exophilic) [27] and that can effectively maintain transmission at lower human LF infection prevalence than other mosquito species [28]. The less common night-biting *Ae. samoanus* is also endemic [29] and serves as a secondary vector. *Culex quinquefasciatus* is also present, but considered of minor importance for LF in the Samoan Islands compared to the *Aedes* vectors [16].

### Survey design

The Surveillance and Monitoring for the Elimination of LF in Samoa (SaMELFS) project conducted human and mosquito surveys before and after both the 2018 and 2023 rounds of triple-drug MDA (Fig 1). The first two surveys occurred in 2018 [30] and 2019 [17,23], and included 35 primary sampling units (PSUs) across Upolu, Savai’i, and Manono Island. In 2023 [25] and 2024, eight PSUs were surveyed for both human-based and mosquito-based indicators. The eight PSUs (S1 Fig) were a subset of those surveyed in 2018 and 2019 and were chosen based on the Ag prevalence in 2019 [25]. The PSUs were selected to represent a range of transmission scenarios resulting in two each being selected from high (13–17%), medium (6–7%), low (3–5%) and zero (0%) observed Ag prevalence in 2019. All eight PSUs were located on Upolu due to logistical and cost constraints. Results from the 2018 survey have been published previously [17,30], and not included in the current study because the human survey occurred immediately after the MDA, meaning no baseline was available for Mf. Furthermore, the 2018 MX survey included only five of the eight PSUs surveyed in 2023 and 2024.

**Fig 1:**
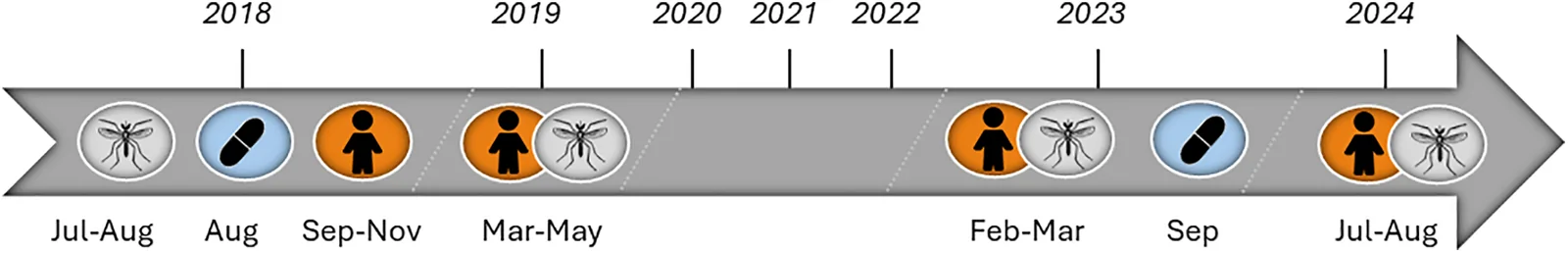
Timeline of Surveillance and Monitoring for the Elimination of Lymphatic Filariasis in Samoa (SaMELFS) human surveys (orange circles) and mosquito surveys (grey circles) in relation to the distribution of triple-drug mass drug administration (blue circles). Dates are provided in S1 Table.

### Human surveys

In each of the eight PSUs, we selected 15 houses using a virtual walk method with a random starting point [30]. Households were approached by a member of the Samoa Red Cross and offered the opportunity to participate in the survey. A household was defined as all people who shared the same kitchen. Household members were eligible to participate if they were aged ≥5 years and had slept at the property within the last week or considered it their main place of residence. Eligibility criteria were consistent over the three surveys. Target sample sizes per PSU were 57 people aged ≥10 years in 2019, 60 people aged ≥5 years in 2023 and 100 people aged ≥5 years in 2024. Specific protocols for the 2019 and 2023 surveys have been described in detail elsewhere [23,25].

Households where all members declined to participate, or where no one was home at the time of the visit, (or after a repeat visit if time allowed) were replaced with the nearest neighbouring household. If the target sample size had not been achieved after 15 houses had been surveyed, additional households were enrolled until the target was met. In 2019, additional houses were selected at random. In 2023 and 2024, respectively, 5 and 10 extra random houses were preselected.

In each survey, all participants (or their guardians) were asked a series of demographic and behavioural questions including whether they participated in the previous MDA round, with their answers recorded electronically by bilingual field teams. A finger-prick blood sample (up to 500 µL) was collected in a heparin microtainer. The samples were kept in cooler bags before being refrigerated in the lab for one (usually) or two nights. Samples were returned to room temperature and tested for LF Ag using Alere/Bioline Filariasis Test Strips (Scarborough, ME, USA) and read at 10 minutes. Two slides of thick blood smears (three rows of 20 µL each) were made from any Ag-positive samples, following WHO guidelines [6]. In 2019 and 2023, both slides from each participant were stained and read under a microscope by two independent readers to identify and count Mf. In 2024, only one set of slides was stained, and the other was read unstained. If the Mf-positivity differed between readers, or if the difference in counts was > 10%, both readers re-examined these slides to reduce the chance that observed discrepancies were due to reader error. Mf density (Mf/mL) was calculated for each Mf-positive participant as the average number of Mf counted from each of the two slides (generally 60 µL per slide) and converted to counts per mL.

### Mosquito surveys

In 2023 and 2024, mosquito surveys were conducted concurrently with the human surveys, with Biogents-Sentinel traps (BG-Sentinel) (Biogents AG, Regensburg, Germany) placed at households within two weeks of the human survey. Where possible, traps were placed at or near participant households from the human survey. BG-Sentinel traps with attractant BG-lures were placed at households for approximately 48 hours, with a mosquito collection and battery change after 24 hours. Mosquitoes were kept in a cooler box until anaesthetised via freezing at -20 °C. Male mosquitoes were discarded and female mosquitoes were pooled by morphological species identification and trap location, with a maximum of 25 mosquitoes per pool. Samples were stored at -20 °C until being preserved. In 2019 and 2023, pooled samples were preserved by oven-drying for three hours at 60 °C. In 2024, mosquito pools were preserved in approximately 200 µL of DNA/RNA shield (Zymo Research, USA, Cat No. R1100-250). Full protocols for the 2023 and 2024 qPCR testing are given in S1 Text. Methods and protocols for the 2019 survey are available in McPherson et al. (2022) [17].

### Statistical analysis

We calculated Ag and Mf prevalence in each of the three surveys (2019, 2023, and 2024), overall for the eight PSUs combined, and individually for each PSU. All prevalence estimates for Ag and Mf were calculated in Stata 18.0 [31] using the ‘svyset’ command to account for the sampling design and probability of selection. Estimates were adjusted for age and sex distribution based on the 2016 census [32]. We also calculated the proportion of Ag-positive participants in each year who were also Mf-positive (potentially infectious). A two-sample test for equality of proportions was used to compare the difference between years. The geometric mean Mf density of Mf-positive participants was calculated for each of the three years and a two-sample t-test on the log-transformed data was used to compare the difference between 2019 and 2024, and between 2023 and 2024.

All estimates of the prevalence of qPCR-positive mosquitoes were calculated in the PoolTestR package [33] using Bayesian mixed effect logistic regression models with adjustments to account for the varying pool size. In the primary analysis by genus, fixed effects for combinations of sample year and genus were used for population-level differences across all sampled sites. For each genus and year, random effects for PSU and trap site (household) were used to account for hierarchical sampling and clustering of infection by location, allowing for possible correlation between random effects for *Aedes* and *Culex* mosquitoes at each location. The degree of clustering (standard deviation of random effects) at the trap and PSU levels were estimated for each mosquito genus but assumed to be constant across sampling years. Prevalence estimates by PSU accounted for uncertainty in generalising to unsampled sites within the PSU.

Overall prevalence estimates across the eight PSUs were calculated by taking the average of estimates for each PSU, i.e., without attempting to generalise to unsampled PSUs. For each PSU, the overall prevalence in both mosquito genera combined was the average of genus-specific estimates, weighted by the number of mosquitoes of each genus tested in that PSU. Estimates for *Ae. polynesiensis* were calculated separately using a model with the same methods but with fixed effects only for year and for each year random effects by PSU and sampling site. Estimates for the prevalence of qPCR-positive mosquitoes are reported with 95% credible intervals (CrI).

To evaluate the impact of the 2023 MDA on LF infection indicators over time, we calculated the odds ratio (OR) of a positive test in 2024 compared to 2019 or 2023. We evaluated all six indicators: three human-based and three MX-based. Human-based indicators (Ag prevalence, Mf prevalence, and proportion of Ag-positive people who were Mf-positive) were calculated using Stata version 18.0 [31]. The PoolTestR package [33] was used to calculate ORs for the mosquito-based indicators (prevalence of qPCR-positive mosquitoes from all species, all *Aedes* species, and *Ae. polynesiensis).*

## Results

### Participant distribution and demographics

In 2024, there were 1,109 participants enrolled. Of these, 1,074 (97%) had valid Ag results, and 35 were missing results due to sample quantity or quality. Participant numbers and demographics for those with valid Ag results are given in Table 1, and results from 2019 and 2023 (published previously [23,25]) have been included for ease of comparison. In the 2024 survey, 924 out of 1074 (86.0%) participants reported taking the medication in the 2023 MDA.

**Table 1:** Participant numbers and demographic details for the 2019, 2023, and 2024 surveys in eight sentinel primary sampling units (PSUs) in Samoa, categorised by 2019 antigen (Ag) prevalence category. Results from 2019 and 2023 have been published previously [23,25] and are included here for comparison. Numbers include only participants with valid Ag results. PSU = primary sampling unit.

Year			2019	2023	2024
Total participants			643	623	1074
Sex n (%)		Male	291 (45.3%)	280 (45.0%)	494 (46.0%)
		Female	352 (54.7%)	343 (55.0%)	580 (54.0%)
Median age in years (range)			20 (5-80)	22 (5-93)	20 (5-94)
Number of households			121	125	187
Number of participants (n, %) by PSU and 2019 Ag prevalence category	Zero (0%)	Vaivase Tai	58 (9.0%)	61 (9.8%)	101 (9.4%)
		Mutiatele & Salea’aumua	89 (13.8%)	68 (10.6%)	132 (12.3%)
	Low (3-6%)	Tuanai	76 (11.8%)	72 (11.6%)	128 (11.9%)
		Fusi	69 (10.7%)	91 (14.6%)	155 (14.4%)
	Medium (6-7%)	Vaiusu	100 (15.6%)	101 (16.2%)	147 (13.7%)
		Falefa	74 (11.5%)	58 (9.3%)	124 (11.5%)
	High (13-16%)	Faleasiu	102 (15.9%)	72 (11.6%)	149 (13.9%)
		Lauli’i	75 (11.7%)	100 (16.1%)	138 (12.8%)

### Antigen prevalence

In 2024, the overall adjusted Ag prevalence in the eight PSUs was 10.3% (95% CI: 7.3-14.5), representing no significant change (p = 0.94) from 2023 when it was 9.9% (95% CI: 3.5-21.0) [25] or from 9.8% (95% CI: 5.6-15.5) in 2019 (Fig 2, S2 Table). Prevalence remained highest in the known hotspots of Faleasiu and Lauli’i.

**Fig 2:**
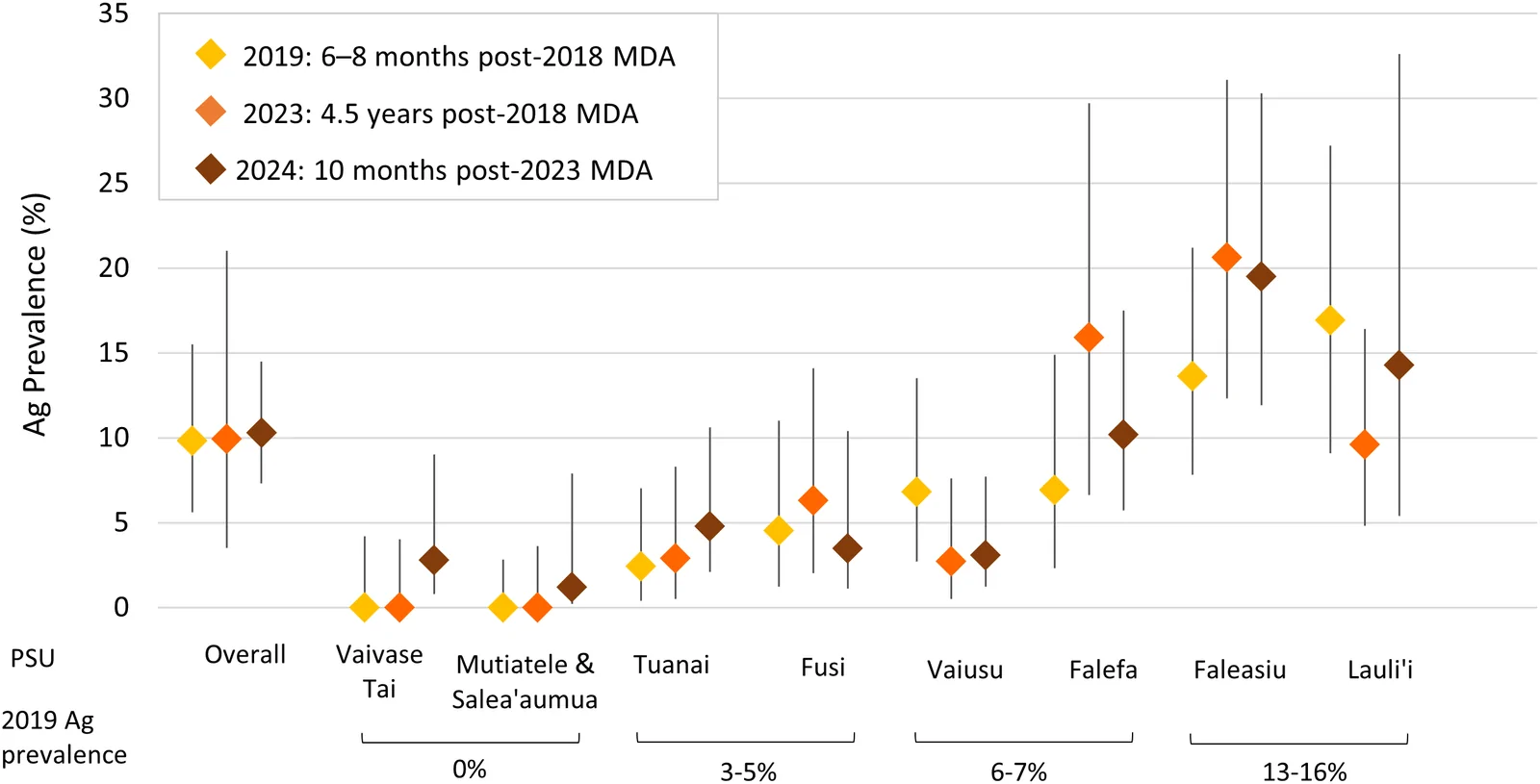
Antigen (Ag) prevalence in Samoa for the eight primary sampling units (PSUs) surveyed in 2019 (6–8 months after the 2018 mass drug administration [MDA]), 2023 (4.5 years after the 2018 MDA) and 2024 (10 months after the 2023 MDA), overall and by PSU. Error bars represent 95% CIs. The 2019 Ag prevalence categories reflect the prevalence from the 2019 survey.

### Microfilaria indicators

In 2024, the overall adjusted Mf prevalence in the eight PSUs was 3.1% (95% CI: 1.8-5.5) compared to 5.1% (95% CI: 1.3-12.4) in 2023 and 3.1% (95% CI: 1.3-5.9) in 2019 (Fig 3, S3 Table). Geometric mean Mf density of the 19 Mf-positive participants in 2024 was 96.7 Mf/mL (range 8.3 to 6633.3). This was not statistically different to previously reported values from the 16 Mf-positive participants in 2023 (108.9 Mf/mL, range 8.3 to 800.0, p = 0.89), or the 11 Mf-positive participants from 2019 (124.2 Mf/mL, range 16.7 to 867.7, p = 0.41). In 2024, 26.5% (95% CI: 16.5-38.6%) of Ag-positive participants were Mf-positive, compared to 40.1% (95% CI: 25.6-57.9%) in 2023 and 31.7% in 2019 (95% CI: 18.1-48.1).

**Fig 3:**
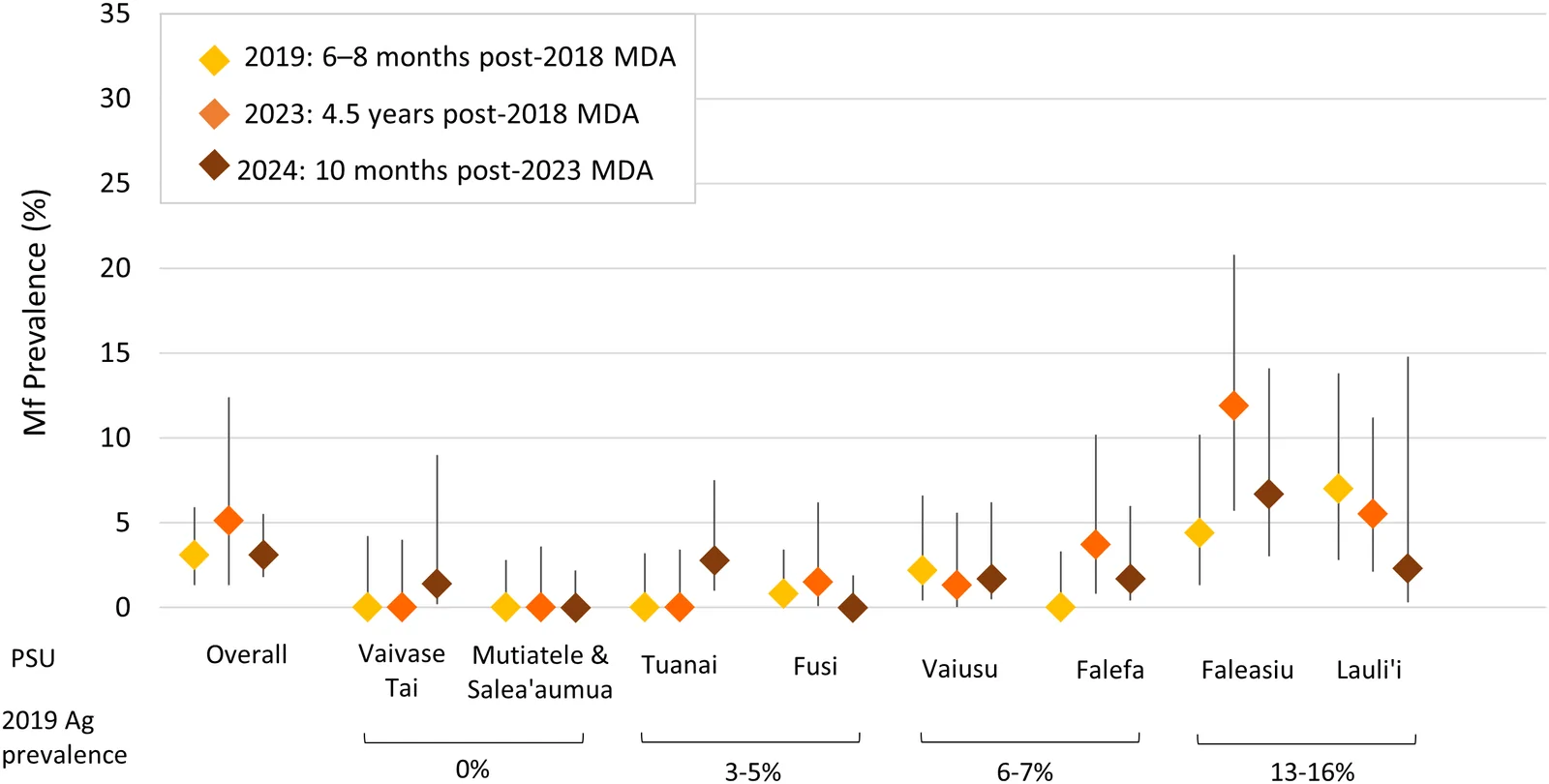
Microfilaria (Mf) prevalence in Samoa for the eight primary sampling units (PSUs) surveyed in 2019 (6–8 months after the 2018 mass drug administration [MDA]), 2023 (4.5 years after the 2018 MDA) and 2024 (10 months after the 2023 MDA), overall and by PSU. Error bars represent 95% CIs. The 2019 Ag prevalence categories reflect the prevalence from the 2019 survey.

### Abundance and species diversity of mosquitoes

In 2023, a total of 5,378 female mosquitoes were tested, grouped into 543 pools (Fig 4, S4 Table). The average catch size per PSU was 672 (range 321–1127). Mean number of mosquitoes per pool was 10.9 (range 1–25) for *Aedes* spp*.* and 5.6 (range 1–25) for *Culex* spp. In 2024, a total of 3,914 female mosquitoes were tested, grouped into 411 pools, with a mean pool size of 10.5 (range 1–25) for *Aedes* spp*.* and 6.8 (range 1–25) for *Culex* spp*.* (S5 Table). The average catch size per PSU was 489 (range 293–644).

**Fig 4:**
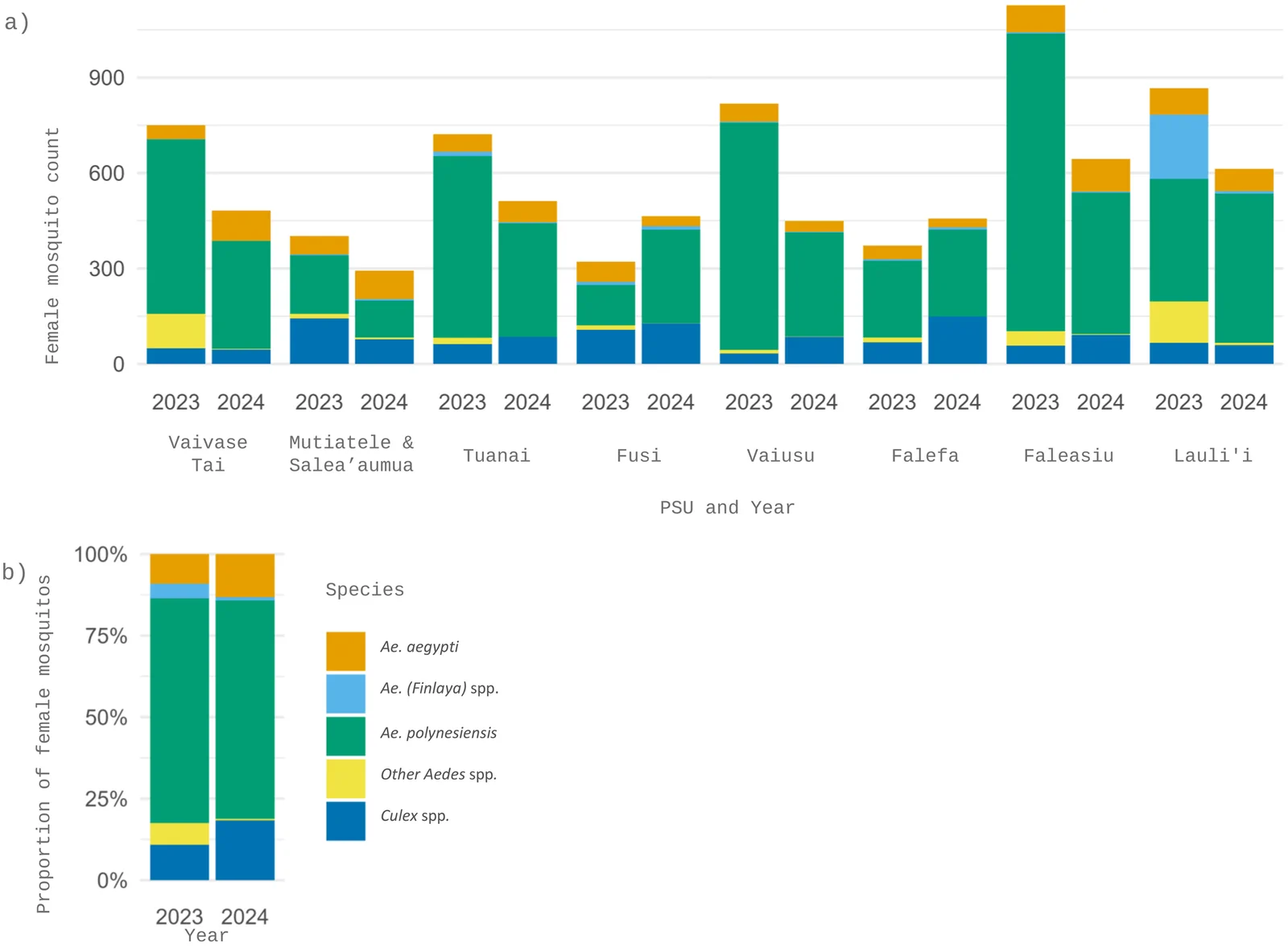
Abundance of female mosquitoes trapped in the Surveillance and Monitoring for the Elimination of LF in Samoa (SaMELFS) 2023 and 2024 MX surveys (a) counts by PSU, and (b) overall proportion by species and year.

*Ae. polynesiensis* was the most common species caught in both 2023 (3,705 mosquitoes, 69% of total catch) and 2024 (2,619 mosquitoes, 67% of total catch). *Culex* mosquitoes made up 11% of the catch in 2023 and 18% in 2024. Species abundance and diversity by PSU are presented in Fig 4. Mean pool sizes and distribution are provided in S5 and S6 Tables. The species distribution in 2019 has also been previously reported [17].

### Prevalence of qPCR-positive mosquitoes

In 2023, 16% (91/543) of all mosquito pools were qPCR-positive. In 2024, 8% (34/411) of all mosquito pools were qPCR-positive. Prevalence of infected mosquitoes was consistently higher in *Aedes* spp*.* compared to *Culex* spp*.* (Fig 5, S7 Table). Estimated prevalence by PSU is given in S2 Fig.

**Fig 5:**
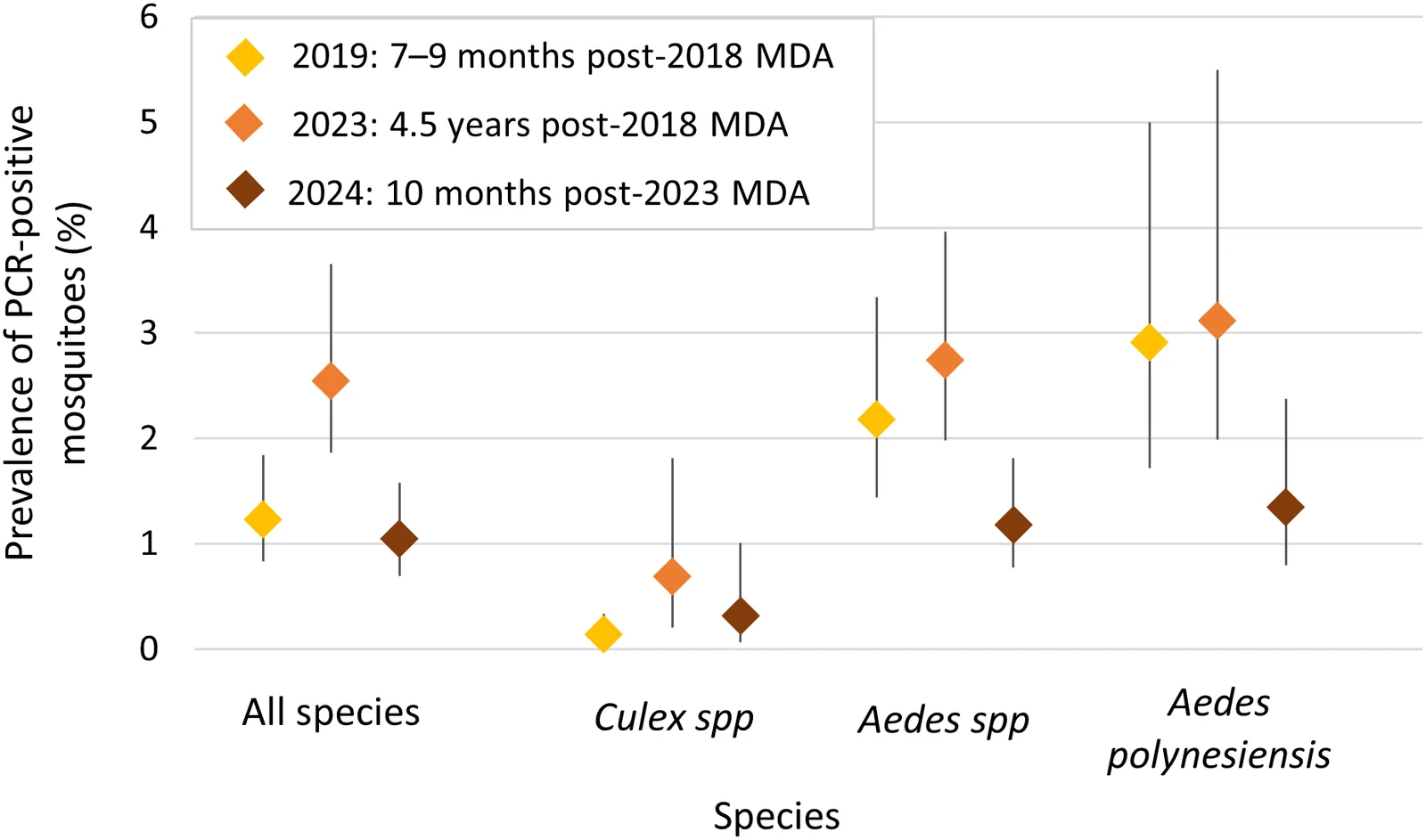
Prevalence of quantitative polymerase chain reaction (qPCR)-positive mosquitoes in eight primary sampling units in Samoa in 2019 (7–9 months after the 2018 mass drug administration [MDA]), 2023 (4.5 years after the 2018 MDA) and 2024 (10 months after the 2023 MDA) for all species, *Culex* spp., all *Aedes* spp., and *Ae. polynesiensis.* Error bars represent the 95% credible intervals.

### Impact of MDA

There was no significant change in Ag prevalence between the 2023 survey (pre-2023 MDA) and the 2024 survey, 10 months after the 2023 MDA (OR 1.0, 95% CI: 0.7, 1.6). Although there were reductions in Mf prevalence from 2023 to 2024 (OR 0.68, 95% CI: 0.33-1.4) and in the proportions of Ag-positives who were Mf-positive, the differences were not statistically significant (Fig 6, S8 Table). For all mosquito-based indicators, ORs showed a decrease in the prevalence of qPCR-positive mosquitoes from 2023 to 2024, and also from 2019 to 2024, noting that in this second time period, the change for all species combined was not statistically significant.

**Fig 6:**
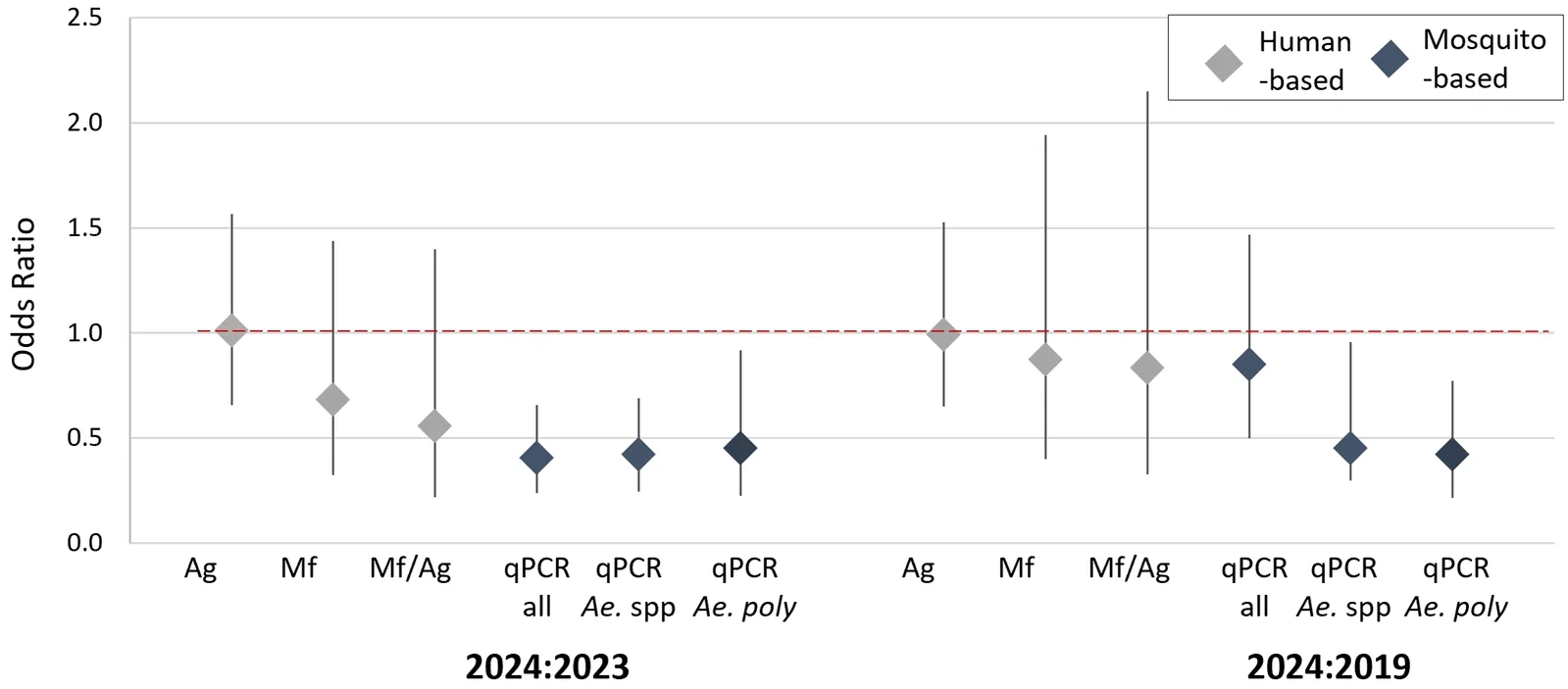
Odds ratios for human and mosquito-based LF indicators in eight primary sampling units in Samoa in 2024 (10 months post-2023 MDA) compared to 2023 (4.5 years post-2018 MDA, pre-2023 MDA), and in 2024 compared to 2019 (6–9 months post-2018 MDA). Human indicators are antigen (Ag), microfilaria (Mf), proportion of Ag-positive people who were also Mf-positive (Mf/Ag). Mosquito indicators are the positive/negative results of quantitative polymerase chain reaction (qPCR)-positive mosquitoes for all species (qPCRall), all *Aedes* species (qPCR *Ae. s*pp.) and *Aedes polynesiensis* (qPCR *Ae. poly*). Horizontal dashed line (OR = 1) indicates no change between years.

## Discussion

Two rounds of triple-drug MDA, spaced five years apart, were not sufficient to break the LF transmission cycle in Samoa. Despite no confirmed reduction in Ag or Mf prevalence following the 2023 MDA round, mosquito-based indicators detected evidence of reduced transmission 10 months after MDA. The non-significant decrease between 2023 and 2024 in both Mf prevalence and the proportion of Ag-positive participants who were Mf-positive (potentially infectious) also represents a likely decrease in transmission intensity. The timeframe needed to detect a similar signal by using Ag alone remains undetermined and it is currently unclear whether countries could expect to see a decrease in Ag prevalence prior to any recommended additional MDA rounds. The relatively small number of Mf-positive participants limits our ability to determine whether the reduction in Mf prevalence represented a statistically significant population-level change. These results therefore do not demonstrate that MX is intrinsically more responsive than Mf. Rather, given the sampling effort used in these surveys, MX provided a more precise and statistically detectable signal of change following MDA compared to Mf.

Based on previous results from Samoa [23], it was unsurprising to find that Ag prevalence had not reduced 10 months after the 2023 MDA. While a single dose of the triple-drug treatment has been shown to be effective at clearing Mf in Samoa within a week [34], Ag can persist in the blood for months or years after infection has been cleared [35]. Evidence from previous surveys in Samoa has shown that recrudescence or reinfection is also possible in those who have been treated [36,37]. The decrease in Mf prevalence 10 months after the 2023 MDA, as well as the decrease in the proportion of Ag-positive people who are potentially infectious (Mf-positive), suggests that at a community level, at least some degree of Mf clearance is maintained over this time. However, if the reduction in Mf prevalence is not enough to reduce transmission, these gains are likely to be lost within five years [25]. It should also be considered that without the MDA, Ag prevalence may have continued to increase, meaning that we cannot be sure if the 2023 MDA slowed or halted an even greater increase in transmission, even if there was no observable decrease from current levels.

Ag results from this study, showing no change 10 months after the intervention, support existing evidence of a delay in Ag reduction following triple-drug MDA [38,39]. Another recent study from Papua New Guinea also found a much larger decrease in Mf prevalence compared to Ag prevalence 12 months after triple-drug MDA [40]. They concluded that Ag was not a suitable indicator for measuring current transmission in regions that have recently distributed MDA. Our results support this assertion: evaluating the impact of an MDA in the early post-intervention period (within 10 months) requires a more time-sensitive measure than Ag.

The mosquito surveillance results from this study support existing evidence from the Pacific, including Samoa [11,17], American Samoa [16], and French Polynesia [14], that MX could strengthen efforts to monitor the impact of MDA in the region. Our results from the 2023 and 2024 surveys in Samoa confirm previous findings from 2018 and 2019 that mosquito-based indicators for filarial DNA are not only useful for providing signals of transmission at specific locations [11], but also serve as a more time-sensitive indicator than Ag for monitoring the impact of MDA in the months immediately after drug distribution [17].

In contrast to recent results from French Polynesia [14] that found the highest prevalence of qPCR-positive mosquitoes in a non-vector species (*Ae. aegypti*), the highest prevalence in our study was seen in the main vector (*Ae. polynesiensis*). Prevalence of qPCR-positive mosquitoes was also consistently higher in *Aedes* spp*.* than in *Culex* spp. This difference was reflected in a lower overall prevalence of qPCR-positive mosquitoes in 2019 (where approximately half the samples were *Culex* spp.) compared to both 2023 and 2024, in which the majority of the catch was *Aedes* spp., raising the overall qPCR-positive prevalence.

In a context where researchers are already challenged with the analytical complexities of estimating prevalence from pooled samples, often with hierarchical survey design [13], whether further complexity should be added by combining genera presents several considerations. Our results found that sorting mosquitoes to species level (all *Aedes* spp. compared to *Ae. polynesiensis*) provided limited additional useful information for decision-makers. However, if genera were combined when comparing prevalence over time, differences in the relative number of each genus tested between timepoints would obscure trends or could create the appearance of changing prevalence where there was none. New tools that simultaneously determine the species composition of a mixed pool and test for the presence of pathogen DNA could remove the need to sort mosquitoes before pooling [41]. However, these methods are yet to be applied to large field surveys and, currently, there is no software available for easily conducting the appropriate statistical analysis of the resulting data. The markedly different prevalence between genera also creates a dilemma when setting targets for MX in a validation setting and raises questions as to whether separate cut-offs are required for different species and regions [13].

There are several limitations to consider when interpreting the results of this study. First, because of the limited sample size of the human surveys, the study lacked statistical power to significantly detect small year-to-year differences in Ag or Mf prevalence. This study compared the sensitivity of human and mosquito surveillance for similar survey effort, i.e., same number of houses in the same villages over two days. We did not design the sample size to compare prevalence in each year to a threshold (e.g., 0.25%) or to have a minimum probability of detecting at least one infected mosquito pool or person for a given prevalence. The consideration for mosquito sample size was the number of mosquitoes needed to detect a statistically significant change (decline or increase) in qPCR positivity prevalence between 2023 and 2024. Despite the lower-than-expected catch sizes for mosquitoes in 2024 due to adverse weather conditions, our results demonstrated a statistically significant decline in prevalence of qPCR-positivity, confirming that a sufficient mosquito sample size was achieved to answer our research question. For detecting differences in Mf or Ag prevalence, our achieved sample size would have had > 70% power to detect declines in prevalence of >50% (assuming a conservative effect size of 2). Declines of this magnitude for Mf prevalence would be reasonably expected if the MDA efficacy and coverage were at levels achieved previously, but the observed decline was smaller than this and not statistically significant. Additionally, although not a limitation, it should be noted that because the sampling design did not involve random selection of villages, overall prevalence estimates in the eight PSUs cannot be generalised to the national level and therefore cannot be considered in relation to the WHO target thresholds [6].

It is possible that the self-reported coverage estimates for the 2023 MDA were higher than the actual achieved coverage. For every population-level intervention there will be a proportion of individuals who did not receive treatment, either by choice or due to accessibility issues. It is likely that these groups are also under-represented in self-reported coverage surveys, leading to an overestimation of actual coverage. Nevertheless, there are several potential explanations for the lack of observed change in the human-based indicators, despite the high reported coverage. For Ag, our results support previous studies on a larger, population representative sample in Samoa that showed no change from the baseline Ag prevalence 7–9 months post-MDA [23]. While the true impact of the MDA cannot be assessed without a counterfactual comparison, the relatively high Ag prevalence in 2024 (10 months post-MDA) could reflect either residual Ag from cleared infections, or ongoing transmission as a result of recrudescence of Mf in treated individuals [36], or ongoing Mf infections in individuals who did not participate, or were not given the correct dose during the 2023 MDA. The Mf prevalence detected in 2024 compared to 2023 is consistent with results from a triple-drug MDA study in Fiji of over 3600 participants, which observed a 62.5% (95% CI, 43.6%–78.2%) efficacy for Mf clearance after 12 months in those treated with triple-drug therapy [42]. The same study found no difference in Mf-clearance between two-drug and triple-drug MDA.

As endemic countries progress towards LF elimination, robust monitoring and surveillance are essential to assess the impact of triple-drug MDA and inform decisions on the need for, and timing of, additional rounds [43]. Ag testing provides a core indicator for identifying populations requiring treatment but has limited sensitivity for detecting short-term changes in transmission following MDA. Mf prevalence may provide earlier evidence of impact than Ag, although its use is constrained by operational and sampling requirements. However, mosquito-based surveillance indicators have demonstrated greater temporal sensitivity to changes in transmission following MDA and can provide more timely programmatic signals than human-based indicators. Clear guidance on the programmatic use of MX is now urgently needed to support standardised survey design, analysis, and interpretation. This integrated surveillance approach, which combines human and mosquito indicators, will be critical to support timely, evidence-based programmatic decision-making to guide adaptive interventions and sustain progress towards interruption of transmission and elimination.

## Supporting information

S1 Fig Selected primary sampling units on the Island of Upolu in Samoa which were surveyed in 2019, 2023, and 2024 as part of the Surveillance and Monitoring for the Elimination of LF in Samoa (SaMELFS) program. (PDF)

S2 Fig PSU-level prevalence of qPCR-positive mosquitoes in the eight primary sampling units in the Surveillance and Monitoring for the Elimination of LF in Samoa (SaMELFS) program surveys in 2019, 2023, and 2024. (PDF)

S1 Text Protocols for 2023 and 2024 qPCR testing of mosquito samples. (PDF)

S1 Table Dates for the Surveillance and Monitoring for the Elimination of LF in Samoa (SaMELFS) program surveys. (PDF)

S2 Table Antigen (Ag) prevalence (%) in Samoa for the eight primary sampling units (PSUs) surveyed in 2019 (6–8 months after the 2018 MDA), 2023 (4.5 years after the 2018 MDA), and 2024 (10 months after the 2023 MDA). (PDF)

S3 Table Microfilaria (Mf) prevalence (%) in Samoa for the eight primary sampling units (PSUs) surveyed in 2019 (6–8 months after the 2018 MDA), 2023 (4.5 years after the 2018 MDA), and 2024 (10 months after the 2023 MDA), overall and by PSU. (PDF)

S4 Table Counts of female mosquitoes trapped by primary sampling units (PSU) in the Surveillance and Monitoring for the Elimination of LF in Samoa (SaMELFS) program surveys in 2019, 2023, and 2024. (PDF)

S5 Table Mean (min-max) number of female mosquitoes per pool in the Surveillance and Monitoring for the Elimination of LF in Samoa (SaMELFS) program surveys in 2019, 2023 and 2024. (PDF)

S6 Table Distribution of female mosquitoes per pool in the Surveillance and Monitoring for the Elimination of Lymphatic Filariasis in Samoa (SaMELFS) program surveys in 2023 and 2024. (PDF)

S7 Table Prevalence of qPCR-positive mosquitoes in eight PSUs in Samoa in 2019 (7–9 months after the 2018 MDA), 2023 (4.5 years after the 2018 MDA) and 2024 (10 months after the 2023 MDA) for all species, *Culex* spp., all *Aedes* spp., and *Ae. polynesiensis.* (PDF)

S8 Table Odds ratios for human and mosquito-based LF indicators in eight primary sampling units in Samoa in 2024 (10 months post-2023 MDA) compared to 2023 (4.5 years post-2018 MDA, pre-2023 MDA), and in 2024 compared to 2019 (6–9 months post 2018-MDA). (PDF)
