# Supplementary material for: Evaluating the impact of triple-drug mass drug administration using human and mosquito-based indicators of lymphatic filariasis in Samoa, 2019 – 2024
Source: PLoS Negl Trop Dis. 2026 Oct 15;20(10):e0013895. doi: 10.1371/journal.pntd.0013895 (PMC13671651; doi:10.1371/journal.pntd.0013895)
Supplement: Supplementary file 1 [file pntd.0013895.s001.pdf]

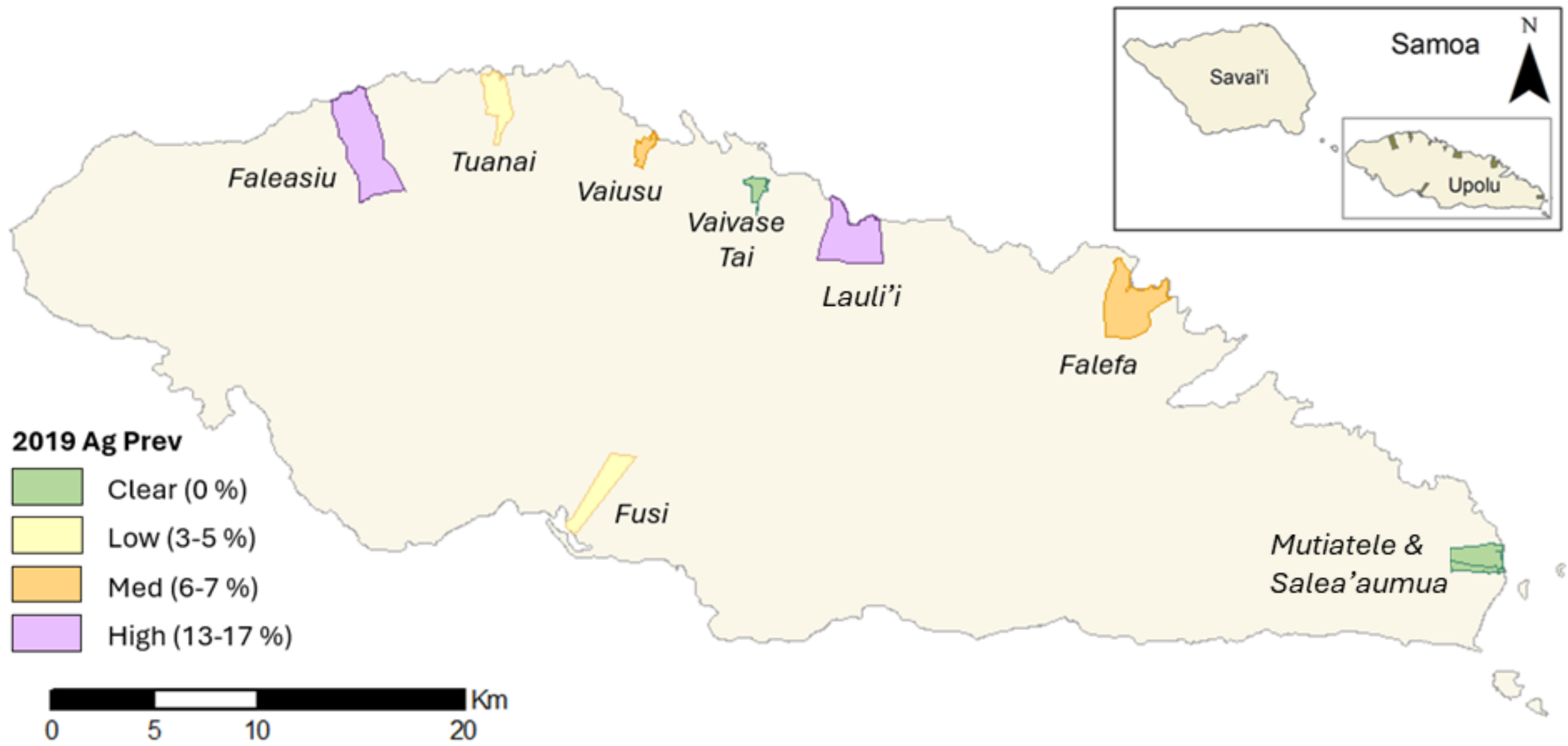

Supplementary Fig S1. Selected primary sampling units on the Island of Upolu in Samoa which were surveyed in 2019, 2023, and 2024 as part of the Surveillance and Monitoring for the Elimination of Lymphatic Filariasis in Samoa (SaMELFS) program. Colour indicates the estimated antigen prevalence at baseline (2019). Spatial data on country, island, region, and village boundaries in Samoa were obtained from the Pacific Data Hub (pacificdata.org accessed on 8 July 2020) and DIVA-GIS (diva-gis.org, accessed on 12 August 2019) under an open access licence available at https://pacific-data.sprep.org/resource/public-data-license-agreement-0.
