# Supplementary material for: Evaluating the impact of triple-drug mass drug administration using human and mosquito-based indicators of lymphatic filariasis in Samoa, 2019 – 2024
Source: PLoS Negl Trop Dis. 2026 Oct 15;20(10):e0013895. doi: 10.1371/journal.pntd.0013895 (PMC13671651; doi:10.1371/journal.pntd.0013895)
Supplement: Supplementary file 2 [file pntd.0013895.s002.pdf]

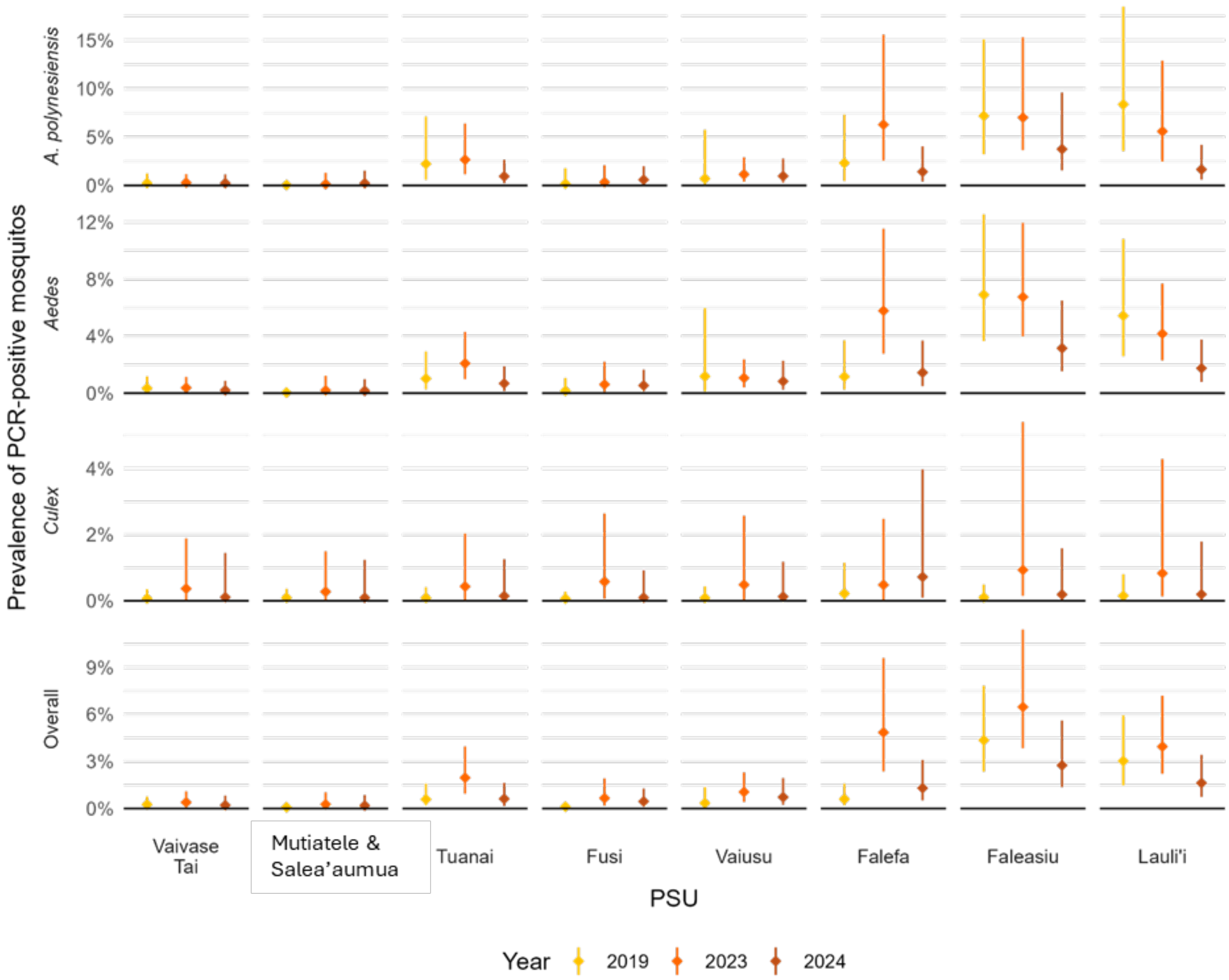

Supplementary Fig. S2. PSU-level prevalence of qPCR-positive mosquitoes in the eight primary sampling units in the Surveillance and Monitoring for the Elimination of LF in Samoa (SaMELFS) program surveys in 2019, 2023, and 2024. Note that y-axes differ by row.
