# Supplementary material for: Evaluating the impact of triple-drug mass drug administration using human and mosquito-based indicators of lymphatic filariasis in Samoa, 2019 – 2024
Source: PLoS Negl Trop Dis. 2026 Oct 15;20(10):e0013895. doi: 10.1371/journal.pntd.0013895 (PMC13671651; doi:10.1371/journal.pntd.0013895)
Supplement: Supplementary file 3 [file pntd.0013895.s003.pdf]

Supplementary Material 1

Protocols for 2023 and 2024 qPCR testing of mosquito samples

2023:

Richard Bradbury & Asrhyella Seeler

Institute of Innovation, Science and Sustainability,
Federation University, Berwik, Victoria, 3806

2024:

Donna MacKenzie, Lisa Rigby, Rhys Izuagbe, Jo Kizu

Australian Defence Force Malaria and Infectious Disease Institute,
Enoggera, Queensland 4051, Australia

2023 qPCR testing protocol

Homogenisation of mosquito tissue in each pool was performed in 1.8 mL Eppendorf tubes (Eppendorf, Hamburg, Germany), with a single 4.5 mm diameter autoclaved steel ballbearing added, in a TissueLyser (Qiagen, Hilden, Germany) at a frequency setting of 30.0 for 10 minutes, or until the macerated tissue material was homogenous. DNA was then extracted from the macerated mosquito tissue using a Qiagen DNeasy Blood and Tissue kit (Qiagen, Hilden, Germany) according to the manufacturer's protocol. A PCR grade water negative control was incorporated into each series of extractions performed.

qPCR was performed on mosquito DNA extracts using the Wb-Cl1 qPCR protocol provided by Quinnipiac University - Pilotte Lab [1]. All qPCR assays were performed using a Realplex 2 Mastercycler (Eppendorf, Hamburg). Each assay consisted of 12.5 µL of PrimeTime® Gene Expression Master Mix (Integrated DNA Technologies, Coraville, IA), 1 µM of forward and reverse primer, 250 nM of TaqMan probe, 6.25 µL of PCR grade water (Integrated DNA Technologies, Coraville, IA) and 5 µL of sample DNA extract. A DNA extract from a previously collected known positive W. bancrofti blood sample and a blank DNA extraction of PCR-grade water were included with each run as positive and negative controls, respectively. Any amplification ≤40 cycles was considered a positive result. Discordant results were repeated a third time, with the consensus of the three assays chosen as the valid result.

[1] Zulch, M.F., N. Pilotte, J.R. Grant, C. Minetti, L.J. Reimer, and S.A. Williams, *Selection and exploitation of prevalent, tandemly repeated genomic targets for improved real-time PCR-based detection of Wuchereria bancrofti and Plasmodium falciparum in mosquitoes.* PLOS ONE, 2020. **15**(5): p. e0232325.

2024 qPCR testing protocol

Homogenisation of mosquito tissue in each pool was performed on a TissueLyser (Qiagen, Hilden, Germany, Cat No. 85600) for 4 minutes at 50 oscillations per second. Pooled mosquito tissue was lysed using the QIAamp® DNA Mini Kit (Qiagen, Cat. No. 51306) according to the manufacturer's instructions. DNA extraction was performed using MagMAX™ Prime Viral/Pathogen Nucleic Acid Isolation Kit (Thermo Fisher Scientific, Waltham, Massachusetts, USA, Cat no. A58145) on an Applied Biosystems MagMax™ Express 96 Magnetic particle processor 710. The total sample lysate (approximately 600 µL) was aliquoted into a deep well 96-sample plate (Thermo Fisher Scientific, Cat no. 950405450). A binding bead mix (285 µL of Binding Solution and 10 µL Total Nucleic Acid Binding Beads per reaction) was added to each sample in the deep well sample plate. The prepared deep well plate was loaded onto the KingFisher™ Flex Purification System and extraction was performed using MagMax_MVP_Standard protocol. Elutions (50 µL) were collected into a KingFisher™ 96-well microplate (Thermo Fisher Scientific, Cat no. 97002540) and sealed with MicroAmp™ Optical Adhesive Film (Applied Biosystems, Thermo Fisher Scientific).

Real-time PCR (qPCR) was performed on mosquito DNA extracts using the Wb-Cl1 qPCR protocol provided by Quinnipiac University - Pilotte Lab. For 50 reactions, 15.6 µL of 10 µM forward primer (GCTGAAAAACATTCGCTTTTGAATG), 10 µM reverse primer (GGGTAATTAAACCGGTGATCT) and 31 µL of 10 µM double quenched probe (56-FAM/ACAACAACT/ZEN/ATATGGGAATGGTGCAGGT/3IABKFQ, Integrated DNA Technologies, Coraville, IA) were added to 625 µL of TaqPath™ Pro Amp™ Master Mix (Applied Biosystems™ Cat A30865) and 312.8 µL of nuclease free water. Each reaction contained 5 µL of DNA template.

Samples were run on a Bio Molecular Systems Magnetic Induction Cycler (MIC) PCR (Bio Molecular Systems, Queensland, Australia) under the following cycling conditions: 50°C for 2 minutes, 95°C for 10 minutes, followed by 40 cycles of 95°C for 15 seconds and 60°C for 1 minute. Samples were classified as qPCR positive if there was amplification within 39 cycles (i.e., C_t <39). Initial qPCR screened positive samples were re-screened in duplicate. Data was analysed on MIC qPCR software v2.10.0.

there were no samples with Ct ≥ 39 and Ct < 40 amongst those processed in 2024 by the Australian Defence Force Malaria and Infectious Disease Institute. The highest Ct that was below 40 was 38.02 (i.e., no pools with Ct ≥39 and <40) and cut off values between the two years were consistent.
