# Supplementary material for: Evaluating the impact of triple-drug mass drug administration using human and mosquito-based indicators of lymphatic filariasis in Samoa, 2019 – 2024
Source: PLoS Negl Trop Dis. 2026 Oct 15;20(10):e0013895. doi: 10.1371/journal.pntd.0013895 (PMC13671651; doi:10.1371/journal.pntd.0013895)
Supplement: Supplementary file 4 [file pntd.0013895.s004.pdf]

Supplementary Table S1. Dates for the Surveillance and Monitoring for the Elimination of LF in Samoa (SaMELFS) program surveys. Triple-drug mass drug administration was implemented by Samoa Ministry of Health from the 14th to the 26th of August 2018 and 16th to 24th September 2023.

Year	Activity	Start date	End date
2018	Mosquito survey	2 Jul	25 Aug
2018	Human survey	26 Sep	09 Nov
2019	Human survey	28 Mar	17 May
2019	Mosquito survey	20 May	6 Jul
2023	Human survey	2 Mar	15 Mar
2023	Mosquito survey	8 Mar	20 Mar
2024	Human survey	25 Jul	17 Aug
2024	Mosquito survey	17 Jul	29 Jul
