# Supplementary material for: Evaluating the impact of triple-drug mass drug administration using human and mosquito-based indicators of lymphatic filariasis in Samoa, 2019 – 2024
Source: PLoS Negl Trop Dis. 2026 Oct 15;20(10):e0013895. doi: 10.1371/journal.pntd.0013895 (PMC13671651; doi:10.1371/journal.pntd.0013895)
Supplement: Supplementary file 6 [file pntd.0013895.s006.pdf]

Supplementary Table S3. Microfilaria (Mf) prevalence (%) in Samoa for the eight primary sampling units (PSUs) surveyed in 2019 (6–8 months after the 2018 MDA), 2023 (4.5 years after the 2018 MDA), and 2024 (10 months after the 2023 MDA), overall and by PSU.

Primary Sampling Unit	2019 (95% CI)	2023 (95% CI)	2024 (95% CI)
**Overall**	**3.1 (1.3-5.9)**	**5.1 (1.3-12.4)**	**3.1 (1.8-5.5)**
Vaivase Tai	0.0 (0.0-4.2)	0.0 (0.0-4.0)	1.4 (0.2-9.0)
Mutiatele & Salea'aumua	0.0 (0.0-2.8)	0.0 (0.0-3.6)	0.0 (0.0-2.2)
Tuanai	0.0 (0.0-3.2)	0.0 (0.0-3.4)	2.8 (1.0-7.5)
Fusi	0.8 (0.1-3.4)	1.5 (0.1-6.2)	0.0 (0.0-1.9)
Vaiusu	2.2 (0.4-6.6)	1.3 (0-5.6)	1.7 (0.5-6.2)
Falefa	0.0 (0.0-3.3)	3.7 (0.8-10.2)	1.7 (0.4-6.0)
Faleasiu	4.4 (1.3-10.2)	11.9 (5.7-20.8)	6.7 (3.0-14.1)
Lauli'i	7.0 (2.8-13.8)	5.5 (2.1-11.2)	2.3 (0.3-14.8)
