# Supplementary material for: Evaluating the impact of triple-drug mass drug administration using human and mosquito-based indicators of lymphatic filariasis in Samoa, 2019 – 2024
Source: PLoS Negl Trop Dis. 2026 Oct 15;20(10):e0013895. doi: 10.1371/journal.pntd.0013895 (PMC13671651; doi:10.1371/journal.pntd.0013895)
Supplement: Supplementary file 7 [file pntd.0013895.s007.pdf]

Supplementary Table S4. Counts of female mosquitoes trapped by primary sampling units (PSU) in the Surveillance and Monitoring for the Elimination of LF in Samoa (SaMELFS) program surveys in 2019, 2023, and 2024. See Fig. 4 in main paper for a visualisation of counts in 2023 and 2024.

**Year**	**Species**	**Primary Sampling Unit**							
		**Faleasiu**	**Falefa**	**Fusi**	**Lauli'i**	**Salea'au mua & Mutiatele**	**Tuanai**	**Vaiusu**	**Vaivase Tai**
2019	*Ae. aegypti*	77	71	154	61	131	149	34	161
	Ae. (Finlaya) spp	4	10	15	34	32	1	4	1
	Ae. polynesiensis	446	146	116	255	683	164	22	362
	Other *Aedes*	21	4	2	17	268	28	-	12
	C. quinquefasciatus	332	457	763	314	928	314	230	327
	Other	3	-	-	1	1	1	-	1
2023	*Ae. aegypti*	85	43	63	83	58	55	57	44
	Ae. (Finlaya) spp	4	5	10	202	3	14	3	1
	Ae. polynesiensis	935	241	127	385	184	571	714	548
	Ae. upolensis *	-	2	-	-	3	-	-	-
	Other *Aedes*	46	13	14	130	11	20	11	108
	C. quinquefasciatus†	56	67	107	66	140	61	33	49
	Other *Culex*†	1	1	-	-	3	1	-	-
2024	*Ae. aegypti*	103	28	31	70	89	67	34	95
	Ae. (Finlaya) spp	3	7	11	8	5	3	2	1
	Ae. polynesiensis	444	273	294	469	116	357	327	339
	Other *Aedes*	3	-	1	7	6	-	1	2
	C. quinquefasciatus†	91	149	127	59	77	85	85	45

* This category is included in 'Other *Aedes*' in Fig 4.

† These categories are collapsed into a single category '*Culex*' in Fig 4.
