# Supplementary material for: Evaluating the impact of triple-drug mass drug administration using human and mosquito-based indicators of lymphatic filariasis in Samoa, 2019 – 2024
Source: PLoS Negl Trop Dis. 2026 Oct 15;20(10):e0013895. doi: 10.1371/journal.pntd.0013895 (PMC13671651; doi:10.1371/journal.pntd.0013895)
Supplement: Supplementary file 8 [file pntd.0013895.s008.pdf]

Supplementary Table S5. Mean (min-max) number of female mosquitoes per pool in the Surveillance and Monitoring for the Elimination of LF in Samoa (SaMELFS) program surveys in 2019, 2023 and 2024.

Primary Sampling Unit	*Aedes*			*Culex*		
	**2019**	**2023**	**2024**	**2019**	**2023**	**2024**
**Overall**	**9.9 (1-25)**	**10.9 (1–25)**	**10.5 (1–25)**	**16.6 (1-25)**	**5.6 (1–25)**	**6.8 (1–25)**
Faleasiu	10.3 (1-25)	14.3 (1–25)	11.5 (1–25)	15.1 (1-25)	4.4 (1–11)	7.6 (1–25)
Falefa	6.8 (1-25)	7.2 (1–25)	9.6 (1–25)	16.9 (1-25)	6.8 (1–25)	9.9 (1–25)
Fusi	7 (1-25)	5.2 (1–21)	9.9 (1–25)	20.6 (3-25)	7.1 (1–20)	7.9 (1–24)
Lauli'i	8.7 (1-25)	11.4 (1–25)	11.8 (1–25)	14.3 (4-25)	5.1 (1–20)	5.4 (1–23)
Salea'aumua & Mutiatele	14.5 (1-25)	6.6 (1–25)	6.4 (1–19)	18.6 (1-25)	9.5 (2–25)	5.9 (1–14)
Tuanai	9.2 (1-25)	11.2 (1–25)	11.5 (1–25)	14.3 (1-25)	4.1 (1–13)	6.1 (1–25)
Vaiusu	2.5 (1-12)	13.5 (1–25)	10.1 (1–25)	12.1 (1-25)	3 (1–11)	6.5 (1–20)
Vaivase Tai	11.9 (1-25)	13 (1–25)	11.8 (1–25)	14.9 (1-25)	3.8 (1–10)	3.8 (1–12)
