# Supplementary material for: Evaluating the impact of triple-drug mass drug administration using human and mosquito-based indicators of lymphatic filariasis in Samoa, 2019 – 2024
Source: PLoS Negl Trop Dis. 2026 Oct 15;20(10):e0013895. doi: 10.1371/journal.pntd.0013895 (PMC13671651; doi:10.1371/journal.pntd.0013895)
Supplement: Supplementary file 9 [file pntd.0013895.s009.pdf]

Supplementary Table S6 Distribution of female mosquitoes per pool in the Surveillance and Monitoring for the Elimination of Lymphatic Filariasis in Samoa (SaMELFS) program surveys in 2023 and 2024.

	Number of pools in 2023 n (%)			***Number of pools in 2024 n (%)***		
**Pool size**	*Ae. polynesiensis*	*Culex* spp.	All *Aedes* spp.	*Ae. polynesiensis*	*Culex* spp.	All *Aedes* spp.
**1**	2 (0.9%)	21 (19.8%)	69 (15.8%)	1 (0.6%)	24 (22.6%)	46 (15.1%)
**2–10**	42 (19.4%)	66 (62.3%)	162 (37.2%)	43 (25.4%)	57 (53.8%)	121 (39.7%)
**11–19**	61 (28.2%)	13 (12.3%)	84 (19.3%)	63 (37.3%)	16 (15.1%)	74 (24.3%)
**≥20**	111 (51.4%)	6 (5.7%)	121 (27.8%)	62 (36.7%)	9 (8.5%)	64 (21.0%)
**Total**	**216**	**106**	**436**	**169**	**106**	**305**
