# Supplementary material for: Evaluating the impact of triple-drug mass drug administration using human and mosquito-based indicators of lymphatic filariasis in Samoa, 2019 – 2024
Source: PLoS Negl Trop Dis. 2026 Oct 15;20(10):e0013895. doi: 10.1371/journal.pntd.0013895 (PMC13671651; doi:10.1371/journal.pntd.0013895)
Supplement: Supplementary file 10 [file pntd.0013895.s010.pdf]

Supplementary Table S8. Odds ratios for human and mosquito-based LF indicators in eight primary sampling units in Samoa in 2024 (10 months post-2023 MDA) compared to 2023 (4.5 years post-2018 MDA, pre-2023 MDA), and in 2024 compared to 2019 (6–9 months post 2018-MDA). Human indicators are antigen, microfilaria, proportion of Antigen-positive people who were also Microfilaria-positive. Mosquito indicators are the positive/negative results of quantitative polymerase chain reaction (qPCR)-positive mosquitoes for all species (All species), All *Aedes* species and *Aedes polynesiensis*

**Survey**	**Indicator**	**Timeframe**	
		**2024-2019**	**2024-2023**
Human	Antigen	0.99 (0.65-1.52)	1.01 (0.66-1.56)
	Microfilaria	0.87 (0.41-1.94)	0.68 (0.33-1.43)
	Microfilaria-positive/ Antigen positive	0.84 (0.33-2.14)	0.56 (0.22-1.39)
Mosquito	qPCR All species	0.85 (0.50-1.46)	0.41 (0.24-0.65)
	qPCR Aedes spp.	0.45 (0.30-0.95)	0.42 (0.25-0.69)
	qPCR Aedes polynesiensis	0.42 (0.22-0.77)	0.45 (0.23-0.91)
